# Effects of sardine-enriched diet on metabolic control, inflammation and gut microbiota in drug-naïve patients with type 2 diabetes: a pilot randomized trial

**DOI:** 10.1186/s12944-016-0245-0

**Published:** 2016-04-18

**Authors:** Mariona Balfegó, Silvia Canivell, Felicia A. Hanzu, Aleix Sala-Vila, Margarita Martínez-Medina, Serafín Murillo, Teresa Mur, Elena G. Ruano, Francisca Linares, Nuria Porras, Silvia Valladares, Maria Fontalba, Elena Roura, Anna Novials, Cristina Hernández, Gloria Aranda, Antoni Sisó-Almirall, Gemma Rojo-Martínez, Rafael Simó, Ramon Gomis

**Affiliations:** CIBER in Diabetes and Associated Metabolic Disorders (CIBERDEM), c/ Monforte de Lemos 3-5 Pabellón 11 planta 0, 28029 Madrid, Spain; Diabetes and Obesity Research Laboratory, August Pi i Sunyer Biomedical Research Institute (IDIBAPS), c/ Roselló 149 planta 5, 08036 Barcelona, Spain; Les Corts Primary Health Care Center, Tranverse Group for Research in Primary Care, IDIBAPS, c/ Mejia Lequerica s/n, 08028 Barcelona, Spain; Department of Endocrinology and Nutrition, Hospital Clínic of Barcelona, c/Villarroel 170 Escala 11 planta 2, 08036 Barcelona, Spain; CIBER in Physiopathology of Obesity and Nutrition (CIBERobn), c/Villarroel 170, Edifici Helios, 08036 Barcelona, Spain; Laboratory of Molecular Microbiology, Biology Department, University of Girona, Av. Montilivi s/n, E-17071 Girona, Spain; Terrassa Sud Primary Health Care Center, Mútua de Terrassa, Av. Santa Eulàlia s/n, 08223 Terrassa, Barcelona Spain; Endocrinology and Nutrition Department, Hospital Carlos Haya, Biomedical Research Institute of Málaga (IBIMA), Plaza Hospital Civil s/n Sótano Pabellón 1, 29009 Málaga, Spain; Vall d’Hebrón Research Institute and Autonomous University of Barcelona, Pg. de la Vall d’Hebrón 119-129 planta 8, Barcelona, Spain; Alicia Foundation, Camí Sant Benet, 08272 Sant Fruitós de Bages, Barcelona, Spain; University of Barcelona, Facultat de Medicina, c/ Casanova 143, 08036 Barcelona, Spain; Present address: Centre Hospitalier Universitaire Vaudois (CHUV), Departement de Endocrinologie, Rue Saint-Martin 3, CH-1003 Lausanne, Switzerland

**Keywords:** Type 2 diabetes, Pilot trial, Nutrition therapy, Oily fish, Sardine

## Abstract

**Background:**

Nutrition therapy is the cornerstone of treating diabetes mellitus. The inclusion of fish (particularly oily fish) at least two times per week is recommended by current international dietary guidelines for type 2 diabetes. In contrast to a large number of human studies examining the effects of oily fish on different cardiovascular risk factors, little research on this topic is available in patients with type 2 diabetes. The aims of this pilot study were to investigate the effects of a sardine-enriched diet on metabolic control, adiponectin, inflammatory markers, erythrocyte membrane fatty acid (EMFA) composition, and gut microbiota in drug-naïve patients with type 2 diabetes.

**Methods:**

35 drug-naïve patients with type 2 diabetes were randomized to follow either a type 2 diabetes standard diet (control group: CG), or a standard diet enriched with 100 g of sardines 5 days a week (sardine group: SG) for 6 months. Anthropometric, dietary information, fasting glycated hemoglobin, glucose, insulin, adiponectin, inflammatory markers, EMFA and specific bacterial strains were determined before and after intervention.

**Results:**

There were no significant differences in glycemic control between groups at the end of the study. Both groups decreased plasma insulin (SG: −35.3 %, *P* = 0.01, CG: −22.6 %, *P* = 0.02) and homeostasis model of assessment - insulin resistance (HOMA-IR) (SG: −39.2 %, *P* = 0.007, CG: −21.8 %, *P* = 0.04) at 6-months from baseline. However only SG increased adiponectin in plasma compared to baseline level (+40.7 %, *P* = 0.04). The omega-3 index increased 2.6 % in the SG compared to 0.6 % in the CG (*P* = 0.001). Both dietary interventions decreased phylum Firmicutes (SG and CG: *P* = 0.04) and increased *E. coli* concentrations (SG: *P* = 0.01, CG: *P* = 0.03) at the end of the study from baseline, whereas SG decreased Firmicutes*/*Bacteroidetes ratio (*P* = 0.04) and increased *Bacteroides-Prevotella* (*P* = 0.004) compared to baseline.

**Conclusions:**

Although enriching diet with 100 g of sardines 5 days a week during 6 months to a type 2 diabetes standard diet seems to have neutral effects on glycemic control in drug-naïve patients with type 2 diabetes, this nutritional intervention could have beneficial effects on cardiovascular risk. Furthermore, both dietary interventions decreased HOMA-IR and altered gut microbiota composition of drug-naïve patients with type 2 diabetes.

**Trial registration:**

Trial number and name of the registry: NCT02294526, ClinicalTrials.gov

**Electronic supplementary material:**

The online version of this article (doi:10.1186/s12944-016-0245-0) contains supplementary material, which is available to authorized users.

## Background

Type 2 diabetes (T2D) is becoming a global epidemic affecting more than 340 millions of people around the world [1]. It is necessary to explore direct and effective approaches in order to minimize and prevent macrovascular and microvascular complications associated with T2D. Nutrition therapy is the cornerstone of treating diabetes mellitus [2], and current international dietary guidelines recommend the inclusion of fish (particularly oily fish) at least two times per week for subjects with T2D [2, 3].

The omega-3 index (O3I) is the eicosapentanoic acid (EPA) + docosahexanoic acid (DHA) content in erythrocytes expressed as a percentage of total identified fatty acids. Besides to be the main determinant of long chain n-3 polyunsaturated fatty acids (LCn-3PUFAs) intake, the O3I is considered as a novel cardiovascular risk factor [4]. The fact that supplements of seafood-derived n-3 (omega 3) failed to improve insulin metabolism in patients with T2D [5] prompted the hypothesis that nutrients other than omega-3 fatty acids may contribute to the beneficial effects of fish in T2D. These nutrients include fish protein and specific amino acids, particularly taurine, an essential amino acid which can be found abundantly in sardines (*Sardina pilchardus*) [6]. In this regard, rats with diabetes and metabolic syndrome improved insulin resistance, inflammatory status and hyperglycemia after a diet supplemented with proteins obtained from sardines [6, 7]. This overall makes sardine consumption an appealing strategy to limit the complications of T2D.

However, the question of whether regular sardine intake without use of oral hypoglycemic agents improves metabolic control and insulin resistance in patients with T2D remains to be explored. To address this issue, we studied the effect of enriching the diet of drug-naïve T2D patients with 100 g of sardines (the average daily consumption of seafood in Spain [8] 5 days a week for 6 months, searching for changes in metabolic control. As secondary endpoints, we assessed changes in erythrocyte membrane fatty acid (EMFA) profile, adiponectin, inflammatory markers and gut microbiota (GM), whose composition is associated to pathological conditions such as obesity and T2D [9].

## Methods

### Subjects

The Pilchardus Study was a multicenter randomized, nutritional pilot trial conducted between October 2012 and November 2013 in free-living men and women who were overweight or obese [body mass index (BMI) between 26 and 35 kg/m^2^]. Candidate subjects were eligible if they had been diagnosed with T2D according to the American Diabetes Association (ADA) criteria [10] and if their glycated haemoglobin (HbA1_c_) levels were between 6.0 and 8.0 % following standard diabetes dietary recommendations. In addition, to be included in the study, participants could not be undergoing treatment with insulin and/or oral antidiabetic drugs, were aged between 40 and 70 year and consumed less than 3 servings of fish per week.

Exclusion criteria were either acute cardiovascular or cerebrovascular events diagnosed during the 2 months prior to randomization; changes in chronic medication during the 3 months prior to randomization; treatment with oral steroids or non steroidal anti-inflammatory drugs for more than 5 days during the month prior to randomization; intake of omega 3 supplements; and allergy to fish or fish protein.

All procedures used in the study protocol were approved by the Clinical Research Ethics Committees of the three study centers involved (Hospital Clínic of Barcelona, Hospital Vall d’Hebron of Barcelona, and Hospital Carlos Haya of Málaga). Written informed consent to participate in the study and to publish individual data were obtained from all subjects prior to the start of the study.

### Study design

The Pilchardus Study consisted of a 2-week lead-in period, followed by a 6-month dietary intervention study. Sixty-two subjects were recruited and screened from the Endocrinology and Nutrition outpatient departments of the three different hospitals (Hospital Clínic, Hospital Vall d’Hebron, Hospital Carlos Haya) and from the Primary Health Care Centres participating in the study. Finally, sixteen men and nineteen women (60.6 ± 1.4 years) were enrolled. Participants were randomly allocated either to sardine group (SG) or to control group (CG). The method used to generate the random allocation sequence was an online randomization software. An external person from the trial staff was in charge of allocating subjects to CG or SG as appropiate.

Participants in the SG (*n* = 19) followed a standard diet for T2D enriched with 100 g of sardines 5 days a week during 6 months. Each subject in SG replaced 100 g of their usual protein foods intake by 100 g of sardines. Participants in the CG (*n* = 16) followed a standard diet for T2D during 6 months without sardine’s supplementation.

The nutritional intervention aimed to control individual carbohydrate portions and modify the quality of dietary protein and fat with no specific caloric restriction, in order to avoid a possible interference of a high weight loss in study results. The only difference between diets in the two intervention groups was that subjects in the SG replaced 100 g of their usual protein foods intake by 100 g of sardines, therefore both dietary interventions had the same nutrient composition except for the type of protein and fat coming from sardines.

Participants in the SG were provided with a free supply of sardines to cover the entire interventional period. This included sardines canned in olive oil and sardine-based products developed by Alícia [11], such as sardine hamburgers, croquettes and paté, all of which contained either 50 or 100 g of canned sardines in olive oil per serving. Moreover, a variety of dishes featuring fresh and canned sardines were developed by Alícia to increase the variety of recipes used in the sardine diet. The canned sardines were given to patients during the monthly visits with the dietitian, and the sardine-based products were sent home, frozen, at the intervention onset and at 3 months.

Dietary intake and dietary habits of all the participants were assessed by a 3-day dietary record at baseline and during the last week of the dietary interventional period. Nutritional composition was analysed by Easy Diet® online software, and total LCn-3PUFAs, EPA + DHA and taurine intakes were analysed by DIAL 1.0 ® software. Nutritional compliance to sardine diet was assessed at the end of dietary intervention by O3I and monthly by Food Frequency Questionnaire (FFQ). Incompliance was defined as consumption of less than 300 g of sardines per week during one month.

**Fig. 1:**
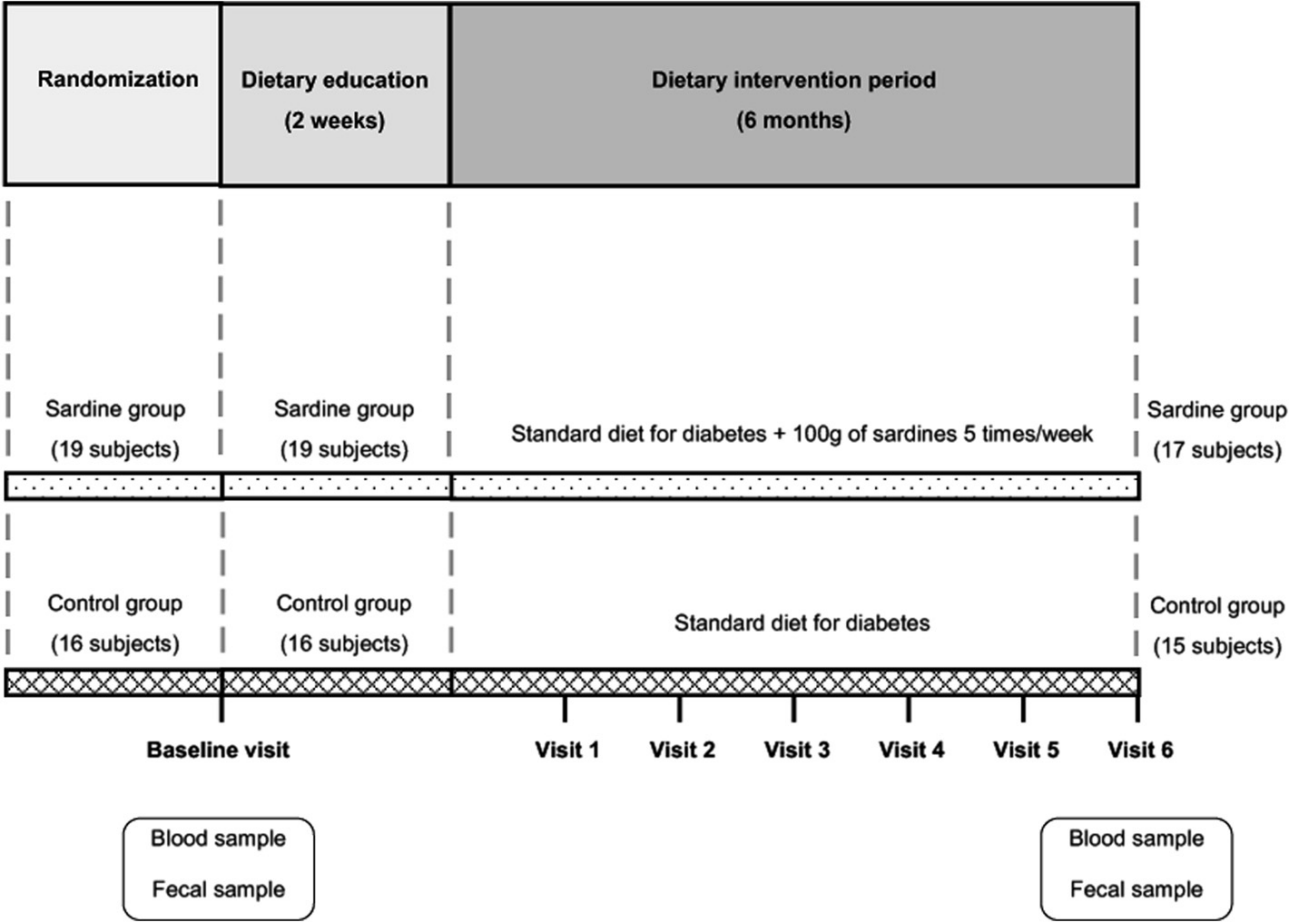
Design of Pilchardus study

During the 2-week lead-in period prior to the start of the nutritional intervention, patients attended two sessions of dietary education. Expert dietitians from the Endocrinology and Nutrition Departments implemented a dietary education program focused on developing the knowledge and skills needed to follow the nutritional prescription used in the study. During the 6-month follow-up period of the study, the same dietitians perfomed monthly follow-up visits with each subject (Fig. 1).

### Clinical measurements and biochemical analysis

At baseline and at the end of the intervention, we obtained anthropometric data and overnight fasted blood samples. Aliquots of blood and plasma were immediately frozen and stored at −80 °C until analysis. All biochemical measurements were analyzed at the Biomedical Diagnostic Center of Hospital Clinic of Barcelona and at the IDIBAPS Diabetes and Obesity Research Laboratory.

For HbA1_c_ determination, an aliquote of whole blood was refrigerated and was analyzed within the first 48 h. Glucose was determined by enzymatic method (ADVIA 2400 Chemistry Analyzer, Siemens Healthcare Diagnostics, Tarrytown, NY), and insulin by chemiluminometric immunoassay (ADVIA Centaur Immunochemistry analyzer, Siemens Healthcare Diagnostics, Tarrytown, NY). Insulin resistance was calculated according to homeostasis model of assessment - insulin resistance (HOMA-IR); HOMA-IR = insulin (mU/ml) × fasting plasma glucose (mmol/l)/22.5, with units expressed as mmol/L. Plasma total adiponectin was determined by commercial ELISA kits (Mercodia AB, Uppsala, Sweden). Plasma interleukin 6 (IL-6) and IL-10 were determined by commercial Cytometric Bead Array (BD™, New Jersey, USA). Plasma tumor necrosis factor alpha (TNF-α), high-sensitivity C-reactive protein (hs-CRP) and IL-8 were determined at the Biomedical Diagnostic Center of Hospital Clinic of Barcelona. Whole blood cell membrane fatty acids were determined by GC (Agilent 6890 Gas Chromatograph HP 6890 with capillary column, autosampler and flame ionization detector) as described [12]. Each fatty acid is expressed as a percentage of total identified fatty acids in the whole blood sample. The O3I was calculated by the sum of percentages of EPA + DHA.

### Gut microbiota composition

Genomic DNA was extracted from fecal samples by using the PSP® Spin Stool DNA Plus kit (Stratec, Berlin, Germany). DNA quantity and purity was checked with NanoDrop ND-100 spectrophotometer. Shifts in microbiota were determined by quantifying the abundance of target bacterial indicators previously associated with T2D by quantitative PCR (qPCR). In particular, we compared the abundance of *Faecalibacterium prausnitzii*, *Escherichia coli (E. coli)*, *Eubacterium rectale – Clostridium coccoides*, *Bacteroides – Prevotella*, Firmicutes and the Firmicutes/Bacteroidetes ratio. (Primers, probes, standards for quantification, and PCR conditions are detailed in Additional file 1).

### Statistical analysis

Descriptive data are presented as the mean or median and standard error (SE) for continuous outcomes, or the number for categorical outcomes. The non-parametric Wilcoxon test for paired data was performed for comparisons between final and baseline values, whereas the U-Mann Withney test was used to compare non-paired data at baseline. Primary and secondary outcomes at 6 months were tested using analysis of covariance (ANCOVA). Statistical analyses were performed using SPSS 18 for Windows (SPSS, Inc., Chicago, IL, USA). *P* values less than 0.05 were considered statistically significant.

## Results

### Baseline subjects characteristics and drop-outs

**Fig. 2:**
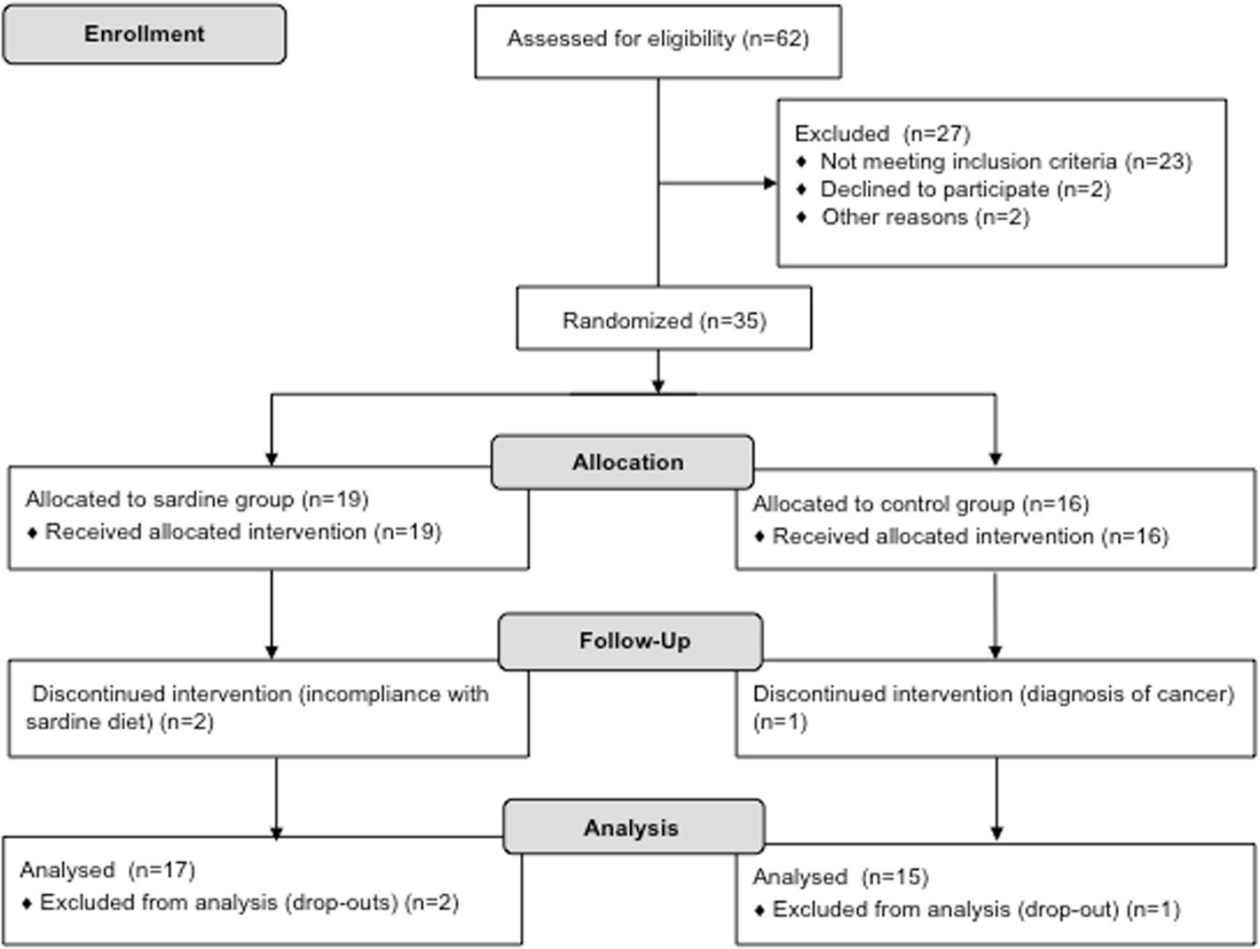
The CONSORT flow diagram

Baseline characteristics of the participants were not significantly different between the two groups (Table 1, Additional file 2). All of them were overweight or obese, had HbA1c values over 6.5 % and had insulin levels between 14.6 and 17.1 mU/L (Table 1). Two subjects of SG were discontinued from the study, both for incompliance with the sardine diet. In the CG, one subject was unable to complete the study due to cancer diagnosis. Of the thirty-five randomized subjects selected to enter the study, a total of thirty-two completed the six-month intervention. Six SG subjects and two CG subjects had no final dietary data; therefore, the changes in dietary intake for these subjects were not included in the analyses performed (Fig. 2).

**Table 1:** Body weight, glycemic, insulin, adiponectin and inflammatory parameters at baseline and after 6 months of dietary intervention

	Baseline			6 Months				
	Sardine group (*n* = 19)	Control group (*n* = 16)		Sardine group (*n* = 17)	Control group (*n* = 15)			
			*P* value^a^			*P* value ^b^	*P* within SG ^c^	*P* within CG ^c^
M^d^/F^e^ (n)	8/11	8/8	0.64	7/10	7/8	0.76		
Age (years)	60.0 ± 1.7	61.2 ± 2.4	0.83	58.7 ± 1.7	59.8 ± 1.9	0.66		
Weight (kg)	81.7 ± 3.7	75.8 ± 2.9	0.25	80.5 ± 3.7	75.1 ± 2.8	0.71	0.08	0.35
BMI^f^ (kg/m^2^)	30.5 ± 1.0	28.8 ± 0.8	0.39	30.0 ± 1.0	28.5 ± 0.8	0.70	0.08	0.44
HbA1c^g^ (%)	6.8 ± 0.1	6.7 ± 0.1	0.59	6.6 ± 0.1	6.4 ± 0.1	0.57	0.08	0.01
Fasting glucose (mg/dL)	138.2 ± 6.5	134.3 ± 5.1	0.99	128.6 ± 4.4	129.1 ± 5.4	0.74	0.18	0.36
Fasting insulin (mU/L)	17.2 ± 1.7	15.1 ± 1.8	0.45	11.1 ± 1.8	11.7 ± 2.4	0.37	0.01	0.02
HOMA-IR^h^	5.9 ± 0.7	5.0 ± 0.6	0.46	3.6 ± 0.6	3.9 ± 0.9	0.34	0.007	0.04
hs-CRP^i^ (mg/dL)	0.2 ± 0.0	0.2 ± 0.1	0.88	0.2 ± 0.0	0.2 ± 0.1	0.73	0.89	0.53
Total adiponectin (μg/mL)	2.1 ± 0.3	2.0 ± 0.4	0.37	3.0 ± 0.3	2.5 ± 0.4	0.26	0.04	0.16
TNFα^j^ (pg/mL)	5.6 ± 0.2	4.9 ± 0.3	0.44	5.6 ± 0.4	6.1 ± 0.5	0.33	0.71	0.05
IL-6^k^ (pg/mL)	3.5 ± 0.3	3.0 ± 0.3	0.66	3.6 ± 0.4	3.3 ± 0.4	0.91	0.68	0.58
IL-8^l^ (pg/mL)	23.3 ± 3.3	18.7 ± 3.0	0.97	25.3 ± 5.3	18.9 ± 1.3	0.50	0.88	0.91
IL-10^m^ (pg/mL)	1.3 ± 0.1	1.2 ± 0.0	0.28	1.5 ± 0.1	1.3 ± 0.1	0.31	0.09	0.12

Values are mean ± SE

^a^ Intergroup comparisons at baseline by Mann-Whitney’s *U* test or *X* ^2^

^b^ Differences between groups at 6 months adjusted for baseline values (ANCOVA)

^c^ Intragroup comparisons by Wilcoxon signed-rank test

^d^ M: male

^e^ F: female

^f^ BMI: Body Mass Index

^g^ HbA1c: glycated hemoglobin

^h^ HOMA-IR: homeostasis model of assessment - insulin resistance

^i^ hs-CRP: high-sensitivity C-reactive protein

^j^ TNFα: tumor necrosis factor alpha

^l^ IL-6: interleukin 6

^l^ IL-8: interleukin 8

^m^ IL-10: interleukin 10

### Body composition and dietary intake

There were no significant changes in body weight between SG and CG at the end of dietary interventions (Table 1). The analyses of 3-day food records showed that the greatest significant differences between groups at 6 months were for nutrients derived from sardine consumption. Total LCn-3PUFA, EPA + DHA and taurine intakes increased significantly in the SG compared to the CG (*P* <0.001) and to baseline (*P* = 0.002) (Table 2). The addition of sardine to the diet provided 19.3 ± 1.0 g of sardine protein, 3.0 ± 0.2 g of EPA + DHA and 118.5 ± 6.2 mg of taurine daily (Additional file 2), which contributed about 23 %, 92 % and 72 % of total protein, EPA + DHA and taurine daily intakes in the SG, respectively (Additional file 3).

**Table 2:** Nutrient dietary intake at baseline and after 6 months of dietary intervention

	Baseline			6 Months				
	Sardine group (*n* = 12)	Control group (*n* = 11)		Sardine group (*n* = 12)	Control group (*n* = 11)			
			*P* value^a^			*P* value ^b^	*P* within SG ^c^	*P* within CG ^c^
Energy (kcal/day)	1829.5 ± 129.9	1758.5 ± 150.7	0.74	1626.2 ± 89.2	1687.2 ± 128.6	0.30	0.02	0.53
Carbohydrates (g/day)	163.8 ± 13.9	146.8 ± 11.7	0.83	141.6 ± 10.8	136.3 ± 14.1	0.82	0.18	0.18
Protein (g/day)	92.0 ± 6.7	87.3 ± 7.9	0.83	83.9 ± 4.8	83.5 ± 5.0	0.85	0.24	0.42
Fat (g/day)	88.3 ± 4.8	84.4 ± 8.1	0.41	79.2 ± 4.5	83.7 ± 5.8	0.27	0.01	0.86
SFA^d^ (g/day)	25.3 ± 2.7	22.6 ± 3.0	0.41	18.6 ± 1.6	21.6 ± 2.2	0.11	0.003	0.72
MUFA^e^ (g/day)	44.8 ± 2.3	42.0 ± 3.3	0.26	40.6 ± 1.9	43.3 ± 2.8	0.25	0.07	0.66
PUFA^f^ (g/day)	11.4 ± 0.6	12.4 ± 1.6	0.93	13.0 ± 1.2	12.0 ± 1.5	0.21	0.21	0.79
EPA^g^ + DHA^h^ (g/day)	0.4 ± 0.1	0.2 ± 0.02	0.15	3.2 ± 0.1	0.4 ± 0.1	<0.001	0.002	0.16
Total n-3 PUFA^i^ (g/day)	1.6 ± 0.2	1.5 ± 0.2	0.60	4.7 ± 0.2	1.3 ± 0.1	<0.001	0.002	0.12
Taurine (mg/day)	61.0 ± 10.9	53.6 ± 7.6	0.95	168.2 ± 9.4	58.7 ± 6.3	<0.001	0.002	0.53
Fat (% Energy)	44.4 ± 1.9	43.1 ± 1.6	0.53	43.8 ± 0.8	44.9 ± 0.9	0.60	0.58	0.25
SFA (% Energy)	12.3 ± 0.8	11.3 ± 0.8	0.49	10.2 ± 0.4	11.3 ± 0.5	0.06	0.03	0.97
MUFA (% Energy)	22.8 ± 1.4	21.9 ± 1.0	0.65	22.7 ± 0.9	23.8 ± 1.2	0.45	0.58	0.13
PUFA (% Energy)	5.8 ± 0.4	6.2 ± 0.4	0.75	7.1 ± 0.5	6.3 ± 0.4	0.01	0.04	0.72
Dietary fiber (g/day)	15.5 ± 1.3	18.9 ± 2.5	0.41	17.3 ± 1.2	15.1 ± 1.5	0.03	0.08	0.08

Values are mean ± SE

^a^ Intergroup comparisons at baseline by Mann-Whitney’s *U* test

^b^ Differences between groups at 6 months adjusted for baseline values (ANCOVA)

^c^ Intragroup comparisons by Wilcoxon signed-rank test

^d^ SFA: saturated fatty acid

^e^ MUFA: monounsaturated fatty acid

^f^ PUFA: polysaturated fatty acid

^g^ EPA: eicosapentanoic acid

^h^ DHA: docosahexanoic acid

^i^ n-3 PUFA: omega 3 polyunsaturated fatty acid

Comparing to baseline, SG patients reduced their caloric (−203.3 ± 82.0 kcal/day, *P* = 0.02) and changed the quality of fat, increasing PUFA (polyunsaturated fatty acid) and decreasing saturated fatty acid (SFA) intake (SFA: −2.2 ± 0.7 % of energy, *P* = 0.03) (Table 2). Regarding to fiber intake, it was observed an increase in the SG compared to the CG (SG: +1.4 ± 1.3 g/day, CG: −3.3 ± 2.0 g/day, *P* = 0.03), while the energy intake or other macronutrient intake did not differ between groups at baseline or at the end of the study (Table 2).

### Glycemic control and insulin sensitivity

The changes in fasting glucose, HbA1c, fasting insulin and HOMA-IR values were similar and non-statistically different between the two intervention diets (Table 1). Only CG decreased significantly Hba1c values compared to baseline (−0.3 % ± 0.1, *P* = 0.01). Both groups significantly reduced fasting insulin and HOMA-IR levels as compared with baseline values, and although the patients in the SG exhibited greater decrease from baseline (SG: −6.1 ± 1.8 mU/L insulin, *P* = 0.01, −2.3 ± 0.7 HOMA-IR, *P* = 0.007, CG: −3.4 ± 1.5 mU/L insulin, *P* = 0.02, −1.1 ± 0.7 HOMA-IR, *P* = 0.04), (Table 1), the mean change from baseline to 6 months was not different between SG and CG (Additional file 4).

### Erythrocyte membrane fatty acid composition

At the end of the intervention, participants in SG increased both EPA (+0.9 ± 0.2 %, *P* = 0.001) and DHA (+1.7 ± 0.2 %, *P* < 0.001), translated into a net significance increase in O3I (+2.6 ± 0.4 %, *P* < 0.001), (Table 3). Despite the decrease in arachidonic acid (AA: C20:4n-6), (−0.9 ± 0.3 %, *P* = 0.02), there was a net increase in total PUFAs (*P* < 0.001). When comparing the two groups, SG showed a significant increase in EPA (*P* = 0.008), DHA (*P* = 0.001), O3I (*P* = 0.001) and total PUFAs (*P* = 0.02) as compared to CG (Table 3).

**Table 3:** EMFA composition at baseline and after 6-month dietary intervention

	Baseline			6 Months				
	Sardine group (*n* = 17)	Control group (*n* = 15)		Sardine group (*n* = 17)	Control group (*n* = 15)			
			*P* value^a^			*P* value ^b^	*P* within SG ^c^	*P* within CG^c^
EPA^d^ (%)	0.6 ± 0.1	0.7 ± 0.1	0.26	1.5 ± 0.2	0.9 ± 0.1	0.008	0.001	0.51
DHA^e^ (%)	4.7 ± 0.2	5.1 ± 0.2	0.35	6.4 ± 0.3	5.5 ± 0.3	0.001	<0.001	0.11
Total n-3 PUFAs^f^ (%)	6.8 ± 0.3	7.5 ± 0.3	0.19	11.6 ± 0.3	8.2 ± 0.5	<0.001	<0.001	0.11
O3I^g^ (%)	5.3 ± 0.3	5.8 ± 0.3	0.26	8.0 ± 0.4	6.4 ± 0.5	0.001	<0.001	0.20
AA^h^ (%)	16.4 ± 0.5	17.2 ± 0.7	0.28	15.4 ± 0.5	17.1 ± 0.6	0.04	0.02	0.39
DHGLA^i^ (%)	1.7 ± 0.1	1.7 ± 0.1	0.63	1.7 ± 0.1	1.7 ± 0.1	0.04	0.34	0.03
Total n-6 PUFAs^j^ (%)	34.1 ± 0.9	34.8 ± 1.0	0.44	32.8 ± 0.9	34.6 ± 0.8	0.12	0.05	0.30
Total n-3 PUFAs + n-6 PUFAs (%)	40.9 ± 0.8	42.2 ± 1.1	0.17	44.4 ± 0.8	42.8 ± 0.7	0.02	<0.001	0.95

Values are mean ± SE

^a^Intergroup comparisons at baseline by Mann-Whitney’s *U* test

^b^ Differences between groups at 6 months adjusted for baseline values (ANCOVA)

^c^ Intragroup comparisons by Wilcoxon signed-rank test

^d^ EPA, eicosapentanoic acid

^e^ DHA, docosahexanoic acid

^f^ n-3 PUFAs: omega 3 polyunsaturated fatty acids

^g^ O3I: omega-3 index

^h^ AA: arachidonic acid

^i^ DHGLA: dihomo-γ-linolenic acid

^j^ n-6 PUFAs: omega 6 polyunsaturated fatty acids

### Total adiponectin and inflammatory markers

Although there were no significant differences in adiponectin concentrations between groups at 6 months (Table 1) or comparing the changes over time (SG: +0.9 ± 0.4, CG: +0.4 ± 0.5, *P* = 0.41) (Additional file 3), those subjects in SG increased significantly the levels of adiponectin from 2.1 ± 0.3 μg/mL at baseline to 3.0 ± 0.3 μg/mL at 6 months (*P* = 0.04) (Table 1). Regarding to inflamatory markers, IL-10 did not show any significant change after the intervention in the SG (baseline: 1.3 ± 0.1 pg/mL, 6 months: 1.5 ± 0.1 pg/mL, *P* = 0.09) (Table 1). In the CG, TNFα concentrations increased significantly when compared to baseline at the end of the study, (*P* = 0.05), (Table 1). Nevertheless, these changes in inflammatory markers were not different between the two intervention groups at 6 months (Table 1).

### Gut microbiota composition

Although no statistically significant differences were found in the abundance of the bacterial groups analysed comparing the SG and the CG at 6 months (Table 4), phylum Firmicutes were decreased in both groups after 6 months (SG: *P* = 0.01, CG: *P* = 0.03) and the Firmicutes*/*Bacteroidetes ratio decreased in SG (*P* = 0.04) (Table 4). *E. coli* concentrations were higher at the end of the study in both SG and CG (SG and CG: *P* = 0.04) (Table 4). Moreover, the proportions of *Bacteroides-Prevotella* increased in the SG (*P* = 0.004), while there was a decreasing trend in the Firmicutes*/*Bacteroidetes ratio in the CG group (*P* = 0.06) (Table 4).

**Table 4:** Gut microbiota composition at baseline and after 6 months of dietary intervention

	Baseline			6 Months				
	Sardine group (*n* = 16)	Control group (*n* = 14)		Sardine group (*n* = 16)	Control group (*n* = 14)			
			*P* value^c^			*P* value ^d^	*P* within SG ^e^	*P* within CG ^e^
Phylum Firmicutes^a^	0.22 ± 0.002	0.22 ± 0.03	0.56	0.16 ± 0.02	0.15 ± 0.01	0.11	0.01	0.03
Phylum Bacteroidetes^a^	0.19 ± 0.03	0.12 ± 0.02	0.19	0.17 ± 0.01	0.18 ± 0.01	0.63	1.00	0.08
Firmicutes/Bacteroidetes ratio^b^	2.59 ± 0.7	1.99 ± 0.5	1.00	1.19 ± 0.1	0.92 ± 0.1	0.75	0.04	0.06
*Bacteroides-Prevotella group* ^a^	0.04 ± 0.006	0.05 ± 0.008	0.56	0.06 ± 0.008	0.06 ± 0.01	0.85	0.004	0.12
*E.rectale-C.coccoides group* ^a, f^	0.09 ± 0.02	0.09 ± 0.02	0.75	0.08 ± 0.02	0.09 ± 0.02	0.97	0.65	0.78
*F.prausnitzii* ^a, g^	0.02 ± 0.003	0.02 ± 0.004	0.73	0.03 ± 0.005	0.02 ± 0.006	0.54	0.11	0.73
*E.coli* ^a, h^	0.0008 ± 0.0002	0.02 ± 0.02	0.93	0.005 ± 0.002	0.08 ± 0.08	0.35	0.04	0.04

Values are mean ± SE

^a^ 16S rRNA gene copies of the bacterial group / 16S rRNA gene copies of total bacteria

^b^ 16S rRNA gene copies of Firmicutes/ 16S rRNA gene copies of Bacteroidetes

^c^ Intergroup comparisons at baseline by Mann-Whitney’s *U* test

^d^ Differences between groups at 6 months adjusted for baseline values (ANCOVA)

^e^ Intragroup comparisons by Wilcoxon signed-rank test

^f^ *E.rectale-C.coccoides*: *Eubacterium rectale – Clostridium coccoides*

^g^ *F.prausnitzii*: *Faecalibacterium prausnitzii*

^h^ *E.coli*: *Escherichia coli*

## Discussion

To our knowledge, the Pilchardus Study is the first study that has been carried out in drug-naïve T2D patients to investigate if a 6-month diet rich in sardines improves glycemic control as compared to a standard diet recommended for T2D. In this sense, this study enhances the field of nutrition research in diabetes.

Previous reports in patients with T2D showed no improvements [13] or small increases in glucose concentrations after a diet rich in fatty fish [14, 15]. Contrasting with these cited studies [14, 15], the Pilchardus Study suggests no deleterious effects of a sardine diet on glycemic control without oral antidiabetic drugs, and during a period of intervention longer enough to observe these changes. In the present study, only the CG reduced significantly its HbA1c levels at the end of the study (− 0.3 % ± 0,1), although the clinical relevance of this value was similar that of the value obtained by the SG (−0.2 % ± 0,1). An important point were that both groups managed to delay, through dietary intervention, the onset of therapy with oral diabetes medication, maintaining a good glycemic control throughout the follow-up period (HbA1c between 6.4 and 6.5 %).

Regarding the reduction of insulin and HOMA-IR in Pilchardus Study, these values were associated with a slight decrease in body weight in both intervention groups, in comparison to the results of other studies, in which weight loss was the main factor [16] or a predictor of improvement in insulin resistance [17]. For this reason, our results differ from the aforementioned studies. In relation to dietary intake, the SG reduced energy intake from baseline but did not significantly modify the body weight. Given that underreporting of energy intake has been previously described in obese patients with T2D [18], this could be a plausible explanation for this observation. In the present study SG and also CG decreased insulin and HOMA-IR levels. The fact that CG had both dietary intervention and dietary education may explain these results, since a variety of approaches in nutrition therapy proves to be effective in improving insulin resistance [2], and dietary education may improve metabolic control inducing modifications in health behaviors [19].

Relating to possible nutrients of oily fish involved in their beneficial effects, because of omega 3 fatty acids supplements failed to yield changes in insulin metabolism in T2D patients [5], some authors emphasized that other nutrients [20], including oily fish protein and taurine (especially in sardines) could have a role in their insulin sensitive effects, as some studies showed in animal models with diabetes and metabolic syndrome [6, 7].

In Pilchardus Study, we could not found a specific effect of oily sardine-enriched diet in insulin resistance, differing from other studies, in which the subjects did not present T2D but healthy weight or overweight/obesity [20, 21]. The reason might be attributed to the differences in metabolic state, given that the studies in subjects with T2D showed the similar results as our study [13], or no modifications in HOMA-IR [14, 15]. It could be possible that effects of oily fish intake in T2D would be dependent on the degree of insulin resistance and glucose deterioration, or the interplay with other factors such as multiple medications, genetic background or macronutrient balance of diet. However, the human evidence of the effects of oily fish on insulin metabolism in T2D is limited to 3 studies [13–15], very scarce comparing to 9 clinical trials conducted with omega 3 supplements; the most recent including 12.350 with T2D [5, 22]. Therefore, the Pilchardus Study enhances the field of nutrition research in diabetes.

In the present study, the inclusion of 100 g of sardines 5 days a week translated to a significant rise in dietary EPA + DHA (3.0 g) and taurine (118.5 mg), those were significantly different from CG. Moreover, the protein and taurine from sardines contributed respectively to 23 % and 72 % of total daily intakes. Therefore, sardine protein and taurine might contribute at some extent to the improvement on HOMA-IR in the SG.

Furthermore, we cannot exclude the possible contributions of olive oil from canned sardines in insulin sensitivity in SG, since and olive oil has favourable effects on insulin sensitivity [23]. Regarding fiber intake, SG exhibited an increase in fiber intake as compared to CG at the end of the dietary intervention. We speculate that the inclusion of sardines during the study could have determined the dietary pattern of patients, so that they included modifications that implied a healthier diet, such as to replace refined cereal products for whole grain cereal products. The evidence indicates that total and cereal fiber is inversely related to the risk of T2D [24], and that a whole-grain diet can improve insulin sensitivity in hyperinsulinemic subjects [25]. Therefore, the increase in fiber intake in SG probably contributed to reduction of HOMA-IR.

As for adipokine profile, in spite that the changes in adiponectin levels was not significant between groups, we observed a 41 % increase in adiponectin levels from baseline values in SG subjects, double the increase found in the CG (21 %). Due to its key role in insulin sensitivity [26], this adipokine might be one of other factors involved in the improvement of insulin resistance in the SG. Some clinical studies have shown the benefits of oily fish intake in adiponectin, but they were not associated with an improvement in HOMA-IR [27, 28]. Perhaps the presence of insulin resistance together with hipoadiponectinemia is needed in order to observe an effect in the improvement on HOMA-IR, as this is a feature of T2D [26] and the cited studies [27, 28] did not include patients with T2D. In addition, since we analysed total adiponectin but not high-molecular weight adiponectin, which is the main isoform that influence adiponectin levels and is related with insulin sensitivity [26], it could be a reason for not finding higher effects in SG.

In respect of oily fish nutrients that can affect adiponectin, both fish oil and taurine have been reported to induce an increase in adiponectin levels in humans [29, 30]. The dose of EPA + DHA from sardines in our study was 3.0 g per day, in the range of those reported in fish oil studies [29], but the dose of taurine supplementation was much higher (3 g per day versus 118.5 mg per day) than in our study [30]. However, because whole foods are provided of superior effects over their isolated constituents [31], these two nutrients might be related to the observed results for adiponectin in SG. Despite this rise in adiponectin, we did not find any modifications in the inflammatory markers studied except for an unexpected rise in TNFα in the CG. However, some studies revealed beneficial effects of oil fish intake on inflammatory markers, such as u-CRP, TNFα and IL-6 [28, 32].

Since the main determinant of O3I is LCn-3PUFAs intake [12], the significant increase of O3I observed in SG attests to its optimal adherence to dietary intervention. Importantly, SG subjects increased the O3I from 5.3 to 8 %, reaching the suggested cutoff for protection against ischemic heart disease, particularly sudden cardiac death [4].

Another remarkable finding related to EMFA composition is the net increase in total PUFAs in red blood cells that was observed in SG. One of the rheological parameters altered in T2D is the deformability of erythrocytes, which has been found to be reduced compared with healthy participants [33]. Such stiffness is likely to reduce microcirculatory flow, impairing diabetes-driven microvascular pathology in the retina and renal glomerulus. In line with this notion, the shift towards insaturation in erythrocite membranes, ensuing increased cell deformability, has been suggested to counteract microangiopathy in T2D [34] Therefore, although sardine intake appears to be unrelated to glycemic control, it might contribute toward limiting vascular co-morbidities associated to T2D.

The Pilchardus Study investigated the impact of a diet rich in sardines on GM in drug-naïve T2D patients, showing a decrease in phylum Firmicutes in both groups and in the Firmicutes/Bacteroidetes ratio in the SG group over time. These results are in agreement with studies in obese patients whose GM was enriched in phylum Firmicutes and the Firmicutes/Bacteroidetes ratio was higher than in control subjects [35]. The opposite was found, however, when they lost weight with low-calorie diets [35] or surgically induced weight loss [36]. There may possibly be an association between the changes in phylum Firmicutes and in Firmicutes/Bacteroidetes ratio with HOMA-IR, as a recent study in mice found and association between a decrease in these bacterial groups and an improvement in insulin resistance [37]. The modification in GM composition in our study could be related to dietary interventions, as shifting dietary macronutrients is proven broadly and rapidly alter the gut microbiome [38]. We speculate a possible effect of LCn-3PUFA present in the sardine diet on GM changes, as certain studies have shown in both mice [39] and infants [40].

In addition, we found increased levels of *E. coli* in both groups and raised proportions of *Bacteroides-Prevotella* in SG patients. Similar results were found in previous studies in obese patients with and without T2D after gastric bypass surgery [36] or following weight loss diets [41]. Although *E. coli* is considered a marker of dysbiosis in T2D [42, 43], only observational studies have established these bacterial imbalances without taking into consideration the diet, which is the main factor affecting GM [9].

It should be noted that we included only drug-naïve T2D patients, thus we can rule out any confounding effect of metformin or insulin on glycemic control or insulin parameters. Moreover, the long duration of the nutritional intervention (6 months) and the use of O3I as a biomarker of LCn-3PUFA confer validity to HbA1c values and to the dietary compliance of the subjects assigned to the SG. In our study, we determined the taurine intake of diets. Given that none of cited studies provided the intake of this sulfur amino acid, the Pilchardus Study is specially novel to this point, contributing to enhance the field of nutrition research in diabetes.

The main limitation of the present study is the small sample size, which makes it difficult to detect changes between the groups due to low statistical power. Another important limitation is the assessment of insulin sensitivity using HOMA-IR as it is a measure of basal insulin sensitivity and does not give information about the stimulated state. In addition, the determination of total adiponectin and not high-molecular weight adiponectin could influence the lack of significant increased in SG compared to CG, considering that this isoform is the most active. Furthermore, the CG also followed a dietary intervention, which could have influenced the results and contributed to producing non-significant results. A third group control, with no intervention at all, or a control group under “usual care” would have been interesting to include, in order to achieve a stronger contrast of results.

## Conclusions

In conclusion, our results suggest that the inclusion of 100 g of sardines 5 days a week during 6 months does not improve glycemic control, but it could have beneficial effects on cardiovascular risk by achieving optimal levels of O3I. Furthermore, both dietary interventions decreased HOMA-IR and altered gut microbiota composition in drug-naïve patients with T2D. Further studies with a greater sample size and extended diet periods would be needed to corroborate our findings and further explore the effects and mechanisms by which a sardine diet could influence metabolic control, adiponectin, inflammation and GM composition in patients with T2D.

## Additional files

Additional file 1: 16S rRNA-targeted primers and probes used in the analysis of gut microbiota. (DOC 56 kb) Additional file 2: Pharmacological treatment of patients at baseline. (DOC 29 kb) Additional file 3: Nutrient intake from sardines at the end of dietary intervention in sardine group. (DOC 35 kb) Additional file 4: Changes from baseline in body weight, glycemia, insulin, adiponectin and inflammatory parameters after dietary intervention. (DOC 39 kb)

## Abbreviations

AA: Arachidonic acid
ADA: American Diabetes Association
ANCOVA: Analysis of covariance
BMI: Body mass index
CG: Control group
DHA: Docosahexanoic acid
EMFA: Erythrocyte membrane fatty acid
EPA: Eicosapentanoic acid
FFQ: Food Frequency Questionnaire
GM: Gut microbiota
HbA1_c_: Glycated hemoglobin
HOMA-IR: Homeostasis model of assessment - insulin resistance
hs-CRP: High-sensitivity C-reactive protein
IL: Interleukin
LCn-3PUFA: Long-chain n-3 polyunsaturated fatty acids
O3I: Omega-3 index
PUFA: Polyunsaturated fatty acid
qPCR: Quantitative PCR
SE: Standard error
SFA: Saturated fatty acid
SG: Sardine group
T2D: Type 2 diabetes
TNF-α: Tumor necrosis factor alpha
