# Supplementary material for: Effects of sardine-enriched diet on metabolic control, inflammation and gut microbiota in drug-naïve patients with type 2 diabetes: a pilot randomized trial
Source: Lipids Health Dis. 2016 Apr 18;15:78. doi: 10.1186/s12944-016-0245-0 (PMC4836051; doi:10.1186/s12944-016-0245-0)
Supplement: Additional file 1: — 16S rRNA-targeted primers and probes used in the analysis of gut microbiota. (DOC 56 kb) [file 12944_2016_245_MOESM1_ESM.doc]

**Additional file 1.** 16S rRNA-targeted primers and probes used in the analysis of gut microbiota

| Target bacteria | Primers and probe*, final concentration at the reaction | Sequence (5'3') | Species used as standard, range of amplification (16S rRNA gene copies) | Ref. |
| --- | --- | --- | --- | --- |
| Total bacteria (*Eubacteria*) | F_Bact 1369, 250 nM | CGGTGAATACGTTCCCGG | *Faecalibacterium prausnitzii* DSM 17677, 10^7^-10^3^ | [1] |
|  | R_Prok1492, 250 nM | TACGGCTACCTTGTTACGACTT |  |  |
|  | **P_TM1389F, 275 nM** | **FAM-CTTGTACACACCGCCCGTC-TAMRA** |  |  |
| *Firmicutes* | Firm934F, 300 nM | GGAGYATGTGGTTTAATTCGAAGCA | *Faecalibacterium prausnitzii* DSM 17677, 10^7^-10^4^ | [2] |
|  | Firm1060R, 300 nM | AGCTGACGACAACCATGCAC |  |  |
| *Bacteroidetes* | Bact934F, 200 nM | GGARCATGTGGTTTAATTCGATGAT | *Bacteroides thetaiotaomicron* VPI 5482, 10^7^-10^2^ | [2] |
|  | Bact1060R, 200 nM | AGCTGACGACAACCATGCAG |  |  |
| *Bacteroides-Prevotella* | F_Bacter 11, 200 nM | CCTWCGATGGATAGGGGTT | *Bacteroides thetaiotaomicron* VPI 5482, 10^6^-10^2^ | [1] |
|  | R_Bacter 08, 200 nM | CACGCTACTTGGCTGGTTCAG |  |  |
|  | **P_Bac303,** 250 nM | **YY-AAGGTCCCCCACATTG-TAMRA** |  |  |
| *Eubacterium rectale - Clostridium coccoides* | ClEubF, 200 nM | CGGTACCTGACTAAGAAGC | *Clostridium coccoides* ATCC 29236, 10^8^-10^3^ | [3] |
|  | ClEubR, 200 nM | AGTTT(C/T)ATTCTTGCGAACG |  |  |
| *Faecalibacterium prausnitzii* | Fpra428F, 300 nM | TGTAAACTCCTGTTGTTGAGGAAGATAA | *Faecalibacterium prausnitzii* DSM 17677, 10^7^-10^3^ | [4] |
|  | Fpra583R, 300 nM | GCGCTCCCTTTACACCCA |  |  |
|  | **Fpra493PR, 250 nM** | **FAM-CAAGGAAGTGACGGCTAACTACGTGCCAG-TAMRA** |  |  |
| *Escherichia coli* | E.coli F, 300 nM | CATGCCGCGTGTATGAAGAA | *Escherichia coli* CECT 105*,* 10^7^-10^3^ | [5] |
|  | E.coli R, 300 nM | CGGGTAACGTCAATGAGCAAA |  |  |
|  | **E.coli P, 100 nM** | **FAM-TATTAACTTTACTCCCTTCCTCCCCGCTGAA-TAMRA** |  |  |
| IAC PCR† | IAC F, 300 nM | TACGGATGAGGAGGACAAAGGA | n.a.^‡^ | [4] |
|  | IAC, 300 nM R | CACTTCGCTCTGATCCATTGG |  |  |
|  | **IAC PR, 250 nM** | **VIC®-CGCCGCTATGGGCATCGCA-TAMRA** |  |  |

^*^Probe sequences are in bold

†IAC: Internal Amplification Control

^‡^ n.a.: not applicable

DNA IAC sequence (5'-3'): TACggATgAggAggACAAAggACgCCgCTATgggCATCgCACCAATggATCAgAgCgAAgTg

**Additional references**

1. Furet J-P, Firmesse O, Gourmelon M, Bridonneau C, Tap J, Mondot S, et al. Comparative assessment of human and farm animal faecal microbiota using real-time quantitative PCR. FEMS Microbiol Ecol. 2009; 68: 351-62.
2. Guo X, Xia X, Tang R, Zhou J, Zhao H, Wang K. Development of a real-time PCR method for Firmicutes and Bacteroidetes in faeces and its application to quantify intestinal population of obese and lean pigs. Lett Appl Microbiol. 2008; 47:367-73.
3. Rinttilä T, Kassinen A, Malinen E, Krogius L, Palva A. Development of an extensive set of 16S rDNA-targeted primers for quantification of pathogenic and indigenous bacteria in faecal samples by real-time PCR. J Appl Microbiol. 2004; 97:1166-77.
4. Lopez-Siles M, Martinez-Medina M, Busquets D, Sabat-Mir M, Duncan SH, Flint HJ, et al. Mucosa-associated Faecalibacterium prausnitzii and Escherichia coli co-abundance can distinguish Irritable Bowel Syndrome and Inflammatory Bowel Disease phenotypes. Int J Med Microbiol IJMM. 2014; 304:464-75.
5. Huijsdens XW, Linskens RK, Mak M, Meuwissen SGM, Vandenbroucke-Grauls CMJE, Savelkoul PHM. Quantification of bacteria adherent to gastrointestinal mucosa by real-time PCR. J Clin Microbiol. 2002; 40:4423-27.
