# Supplementary material for: Effects of sardine-enriched diet on metabolic control, inflammation and gut microbiota in drug-naïve patients with type 2 diabetes: a pilot randomized trial
Source: Lipids Health Dis. 2016 Apr 18;15:78. doi: 10.1186/s12944-016-0245-0 (PMC4836051; doi:10.1186/s12944-016-0245-0)
Supplement: Additional file 2: — Pharmacological treatment of patients at baseline. (DOC 29 kb) [file 12944_2016_245_MOESM2_ESM.doc]

**Additional file 4.** Pharmacological treatment of patients at baseline

|  | **Sardine group**  **(n=19)** | **Control group**  **(n=16)** | ***P* value**^*^ |
| --- | --- | --- | --- |
| Statins (n) | 2 | 3 | 0.88 |
| Beta blockers (n) | 1 | 3 | 0.82 |

**^*^** Intergroup comparisons at baseline by X^2^
