# Supplementary material for: Effects of sardine-enriched diet on metabolic control, inflammation and gut microbiota in drug-naïve patients with type 2 diabetes: a pilot randomized trial
Source: Lipids Health Dis. 2016 Apr 18;15:78. doi: 10.1186/s12944-016-0245-0 (PMC4836051; doi:10.1186/s12944-016-0245-0)
Supplement: Additional file 3: — Nutrient intake from sardines at the end of dietary intervention in sardine group. (DOC 35 kb) [file 12944_2016_245_MOESM3_ESM.doc]

**Additional file 2.** Nutrient intake from sardines at the end of dietary intervention in sardine group

|  | **Absolute intake**  **(n=12)** | **Relative intake (%)**  **(n=12)** |
| --- | --- | --- |
| Energy (kcal/day) | 175.6 ± 9.2 | 11.1 ± 0.7 |
| Protein (g/day) | 19.3 ± 1.0 | 23.5 ± 1.5 |
| Fat (g/day) | 11.0 ± 0.6 | 14.3 ± 1.2 |
| SFA^1^ (g/day) | 2.0 ± 0.1 | 11.2 ± 1.1 |
| MUFA^2^ (g/day) | 3.8 ± 0.2 | 9.6 ± 0.8 |
| PUFA^3^ (g/day) | 3.9 ± 0.2 | 32.9 ± 3.2 |
| Total n-3 PUFA^4^ (g/day) | 3.5 ± 0.2 | 73.8 ± 2.8 |
| EPA^5^ +DHA^6^ (g/day) | 3.0 ± 0.2 | 92.3 ± 2.8 |
| Taurine (mg/day) | 118.5 ± 6.2 | 72.0 ± 3.7 |

Values are mean ± SE

^1^ SFA: saturated fatty acid

^2^ MUFA: monounsaturated fatty acid

^3^ PUFA: polysaturated fatty acid

^4^ n-3 PUFA: omega 3 polyunsaturated fatty acid

^5^ EPA: eicosapentanoic acid

^6^ DHA: docosahexanoic acid
