# Supplementary material for: Effects of sardine-enriched diet on metabolic control, inflammation and gut microbiota in drug-naïve patients with type 2 diabetes: a pilot randomized trial
Source: Lipids Health Dis. 2016 Apr 18;15:78. doi: 10.1186/s12944-016-0245-0 (PMC4836051; doi:10.1186/s12944-016-0245-0)
Supplement: Additional file 4: — Changes from baseline in body weight, glycemia, insulin, adiponectin and inflammatory parameters after dietary intervention. (DOC 39 kb) [file 12944_2016_245_MOESM4_ESM.doc]

**Additional file 3.** Changes from baseline in body weight, glycemia, insulin, adiponectin and inflammatory parameters after the dietary intervention

Values are mean ± SE

|  | **Sardine group**  **(n=19)** | **Control group**  **(n=16)** | ***P* value**^*^ |
| --- | --- | --- | --- |
| Weight (kg) | -1.2 ± 0.6 | -0.7 ± 0.7 | 0.74 |
| BMI (kg/m^2^) | -0.5 ± 0.2 | -0.2 ± 0.3 | 0.68 |
| HbA1c (%) | -0.2 ± 0.1 | -0.3 ± 0.1 | 0.35 |
| Fasting glucose (mg/dL) | -9.6 ± 5.4 | -5.2 ± 5.5 | 0.97 |
| Fasting insulin (mU/L) | -6.1 ± 1.8 | -3.4 ± 1.5 | 0.60 |
| HOMA-IR | -2.3 ± 0.7 | -1.1 ± 0.7 | 0.60 |
| hs-CRP (mg/dL) | -0.02 ± 0.2 | -0.01 ± 0.2 | 0.75 |
| Adiponectin (μg/mL) | +0.9 ± 0.4 | +0.4 ± 0.5 | 0.41 |
| TNFα (pg/mL) | -0.1 ± 0.4 | +1.2 ± 0.5 | 0.10 |
| IL-6 (pg/mL) | +0.1 ± 0.3 | +0.3 ± 0.4 | 0.98 |
| IL-8 (pg/mL) | +2.0 ± 4.8 | +0.1 ± 3.3 | 0.97 |
| IL-10 (pg/mL) | +0.2 ± 0.1 | +0.1 ± 0.1 | 0.56 |

**^*^** Intergroup comparisons by Mann-Whitney’s U test
